# Myocardial infarction elevates endoplasmic reticulum stress and protein aggregation in heart as well as brain

**DOI:** 10.1007/s11010-023-04856-3

**Published:** 2023-11-03

**Authors:** Nirjal Mainali, Xiao Li, Xianwei Wang, Meenakshisundaram Balasubramaniam, Akshatha Ganne, Rajshekhar Kore, Robert J. Shmookler Reis, Jawahar L. Mehta, Srinivas Ayyadevara

**Affiliations:** 1 Bioinformatics Program, University of Arkansas at Little Rock and University of Arkansas for Medical Sciences, Little Rock, AR 72205 USA; 2 Department of Geriatrics and Reynolds Institute on Aging, University of Arkansas for Medical Sciences, Little Rock, AR 72205 USA; 3 Central Arkansas Veterans Healthcare Service, Little Rock, AR 72205 USA; 4 Division of Cardiology, University of Arkansas for Medical Sciences and Central Arkansas Veterans Healthcare System, Little Rock, AR USA; 5 Henan Key Laboratory of Medical Tissue Regeneration, Xinxiang Medical University, Xinxiang, 453003 China; 6 Department of Cardiology, University of Arkansas for Medical Sciences and Central Arkansas Veterans Healthcare System, Little Rock, AR USA

**Keywords:** Myocardial infarction (MI), Protein aggregation, Endoplasmic reticulum stress (ER stress), Hypoxia, Exosomes, Alzheimer’s disease, Aging

## Abstract

Cardiovascular diseases, including myocardial infarction (MI), constitute the leading cause of morbidity and mortality worldwide. Protein-aggregate deposition is a hallmark of aging and neurodegeneration. Our previous study reported that aggregation is strikingly elevated in hearts of hypertensive and aged mice; however, no prior study has addressed MI effects on aggregation in heart or brain. Here, we present novel data on heart and brain aggregation in mice following experimental MI, induced by left coronary artery (LCA) ligation. Infarcted and peri-infarcted heart tissue, and whole cerebra, were isolated from mice at sacrifice, 7 days following LCA ligation. Sham-MI mice (identical surgery without ligation) served as controls. We purified detergent-insoluble aggregates from these tissues, and quantified key protein constituents by high-resolution mass spectrometry (LC–MS/MS). Infarct heart tissue had 2.5- to 10-fold more aggregates than non-infarct or sham-MI heart tissue (each *P* = 0.001). Protein constituents from MI cerebral aggregates overlapped substantially with those from human Alzheimer’s disease brain. Prior injection of mice with mesenchymal stem cell (MSC) exosomes, shown to limit infarct size after LCA ligation, reduced cardiac aggregation ~ 60%, and attenuated markers of endoplasmic reticulum (ER) stress in heart and brain (GRP78, ATF6, P-PERK) by 50–75%. MI also elevated aggregate constituents enriched in Alzheimer’s disease (AD) aggregates, such as proteasomal subunits, heat-shock proteins, complement C3, clusterin/ApoJ, and other apolipoproteins. These data provide novel evidence that aggregation is elevated in mouse hearts and brains after myocardial ischemia, leading to cognitive impairment resembling AD, but can be attenuated by exosomes or drug (CDN1163) interventions that oppose ER stress.

**Supplementary Information:**

The online version contains supplementary material available at 10.1007/s11010-023-04856-3.

## Introduction

Narrowing of arteries due to plaque buildup reduces blood flow to the affected regions; thrombosis in narrowed atherosclerotic coronary arteries leads to myocardial infarction (MI) [1], which impairs cardiac function [2, 3]. Cardiomyocyte death following MI can promote fibroblast growth and collagen deposition resulting in diastolic dysfunction, and ultimately to systolic heart failure [4], to which other factors may also contribute [1]. Intracellular protein aggregation is frequently seen in aged and hypertensive hearts [5], as well as in brains of patients with neurodegenerative diseases such as Alzheimer’s disease (AD) [6]. However, the impact of MI on protein aggregation in heart and brain has received little or no attention.

Aging alters the synthesis, folding, processing, and degradation of proteins [7], and deficiencies in protein quality control can lead to age-dependent aggregate accrual [5]. Protein aggregates are cytotoxic, for reasons that are uncertain but may include membrane permeabilization [8], oxidative and ER stress [9], and mitochondrial dysfunction [10]. Genetic disruption of any of these pathways can lead to heritable cardiomyopathies that feature increased aggregate deposition.

In the ubiquitin–proteasome system (U/P-S), E3 protein ligases recognize specific misfolded proteins to initiate their polyubiquitin tagging for degradation by proteasomes [11]. Protein quality control systems, comprising both the U/P-S and autophagy, are compromised by myocardial infarction and other cardiovascular diseases (CVDs) [12]. Dysglycemia is associated with higher mortality in patients with MI, possibly acting via autophagy pathways [13]. Hyperglycemia occurs early in the course of type-2 diabetes (T2DM) and promotes both proteasomal activation and NF-kB-mediated inflammatory responses [14]. Islet amyloid polypeptide, or amylin, is a 37-amino-acid peptide secreted by the pancreas. It forms pancreatic amyloid deposits in diabetics [15], although their toxic form is normally cleared by autophagy [16]. All these changes increase vulnerability to plaque rupture and raise the severity of MI and heart failure in diabetic patients [17].

Changes in cognitive function are often observed in patients with coronary risk factors, and in particular among those who have suffered an MI [18]. It has been suggested that MI impairs brain ultrastructure but it is not known if the mechanism is MI induction of protein aggregation in the brain, thereby damaging neurons and impairing cognitive function.

We isolated, identified, and quantified protein constituents of detergent-insoluble aggregates in heart and brain, to clarify the role of myocardial ischemia in protein aggregation. In parallel, we studied endoplasmic reticulum (ER) stress pathways in the mouse heart, because ER stress is intimately associated with protein aggregation. Finally, we examined the effects of mesenchymal stem cell (MSC) exosomes (membranous vesicles generated by almost all cells), which have been shown to decrease apoptosis, inflammation, and infarct size, and to assist in post-MI recovery of cardiac function [19].

## Methods

### Mouse model of sustained ischemia

Adult male C57BL/6J mice were obtained from the Jackson Laboratory (Bar Harbor, ME). The animal study protocol was approved by the institutional Animal Care & Use Committee and conformed to the Guide for the Care and Use of Laboratory Animals (National Academies Press, Washington DC). Mice were euthanatized by CO_2_ inhalation, 7 days after left coronary artery (LCA) ligation.

Myocardial infarction (MI) was induced by total LCA occlusion in anesthetized mice as described previously [20]. In brief, after intraperitoneal injection of ketamine hydrochloride (60 mg/kg) and xylazine hydrochloride (8 mg/kg), anesthetized mice underwent endotracheal intubation and were mechanically ventilated (1.2 ml/min tidal volume, with respiration rate ~ 110/min). For LCA ligature, an 8–0 silk suture was passed around the LCA at a point two-thirds of the way from its origin near the pulmonary conus. The thoracic cavity was closed in layers, using 6–0 sutures, and drained to prevent pneumothorax. Another group of animals underwent the same procedure, but without LCA occlusion (sham ischemia, or sham-MI). A third group of mice was pretreated with a single dose of MSC exosomes (0.5 mg/kg body weight, administered intravenously though the lateral tail vein), 30 min prior to LCA ligation. The requisite quantity of MSC exosomes administered to mice was based on the results of previous studies [21]. Infarcted and non-infarcted heart tissues were excised post-mortem from simulated-MI mice, as described previously [20, 22] and cerebra were also removed, 7 days after LCA ligation.

### Culture of mesenchymal stem cells, and exosome isolation and characterization

An MSC cell line, derived from human bone marrow obtained from a healthy donor and immortalized, was a gift from Dr. Robert J. Griffin at UAMS. MSCs were cultured in growth medium (DMEM; ThermoFisher Scientific, Waltham MA) containing 10% v/v fetal bovine serum (FBS; Atlanta Biologicals Inc., Flowery Branch GA) and 100 units/ml each of penicillin and streptomycin (ThermoFisher), in an incubator maintaining 5% CO_2_ at 37 °C. After overnight culture, MSCs were washed with PBS and covered with fresh serum-free DMEM as described [21]. Exosomes were isolated from medium collected from these cells after a further 16–18 h of incubation at 37 °C in a 5% CO_2_ incubator.

Exosomes were recovered by differential centrifugation at 4 °C as previously described [23]. In brief, medium from MSC cultures was centrifuged 10 min at 3000×*g*, and then 30 min at 10,000×*g*. The second supernatant was centrifuged 3 h at 100,000×*g* to purify exosomes. The exosome pellet was resuspended in PBS, and recovered by centrifugation for 2 h at 100,000×*g*. The final pellet was resuspended in PBS.

MSC exosomes were imaged using a JEOL JSM7000F scanning electron microscope as previously described [24]. Isolated vesicles were characterized as exosomes based on western blots with antibodies to two markers specific for exosomal vesicles (from System Biosciences): CD9 (#EXOAB-CD9A-1) and CD63 (#EXOAB-CD63A-1).

### Aggregate protein isolation

Tissues were minced and homogenized with mortar and pestle at 0 °C in lysis buffer: 20-mM HEPES buffer pH 7.4, 0.3-M NaCl, 2-mM MgCl_2_, 1% (w/v) NP40, containing inhibitors of proteases and phosphatases [CalBiochem]. Homogenates, after sonication on ice (3 × 10 s), were centrifuged 5 min at 2000×*g* to remove debris. Lysate protein concentrations were determined (Bradford Assay, Bio-Rad). After 15-min centrifugation at 14,000×*g*, pellets containing aggregates were resuspended in 0.1-M HEPES buffer, 1% sarcosyl (v/v) and 5-mM EDTA, and centrifuged 30 min at 100,000×*g*. Pellets and supernatants (detergent-insoluble and -soluble fractions, respectively) were resuspended in Laemmli buffer at 95°C and electrophoresed on polyacrylamide gels; 1-mm slices were incubated in trypsin for LC–MS/MS analysis as described [6].

### Thioflavin T staining of cultured human glioblastoma (T98G) cells

Human glioblastoma cells were cultured to 80% confluence in 100-mm Petri dishes at 37 °C. Cells were trypsinized and replated at 6000‒8000 cells/well in 96-well plates, and grown for 16 h at 37 °C in “DMEM + F12” (Life Technologies) medium supplemented with 10% (v/v) fetal bovine serum. A “normoxic” plate was maintained in a CO_2_ incubator with 20% oxygen. A “hypoxic” plate was placed in a hypoxia chamber circulating a gas mixture of 5.1% CO_2_ and 94.9% N_2_ (Nexair cat# SG CO_2_/NI-20); the chamber was flushed twice within 20 min, and the plate was then transferred to a 37 °C incubator with 0% humidity and no gas input for 7 h at 37 °C. After this hypoxic interval, both plates were returned to a normoxic incubator maintaining 5% CO_2_ and 95% air (20% O_2_) at 37 °C, simulating abrupt reperfusion of cells. At this time CDN1163 was added to the treatment wells at 10 µM. After 48 h of further culture, amyloid and similar aggregates were stained with Thioflavin T as reported previously [25]. Briefly, cells were fixed 15 min in formaldehyde (4% v/v), washed, and stained 20 min in a dark container with 0.1% w/v Thioflavin T mixed with DAPI (1 µg/ml; Life Technologies, Grand Island NY). After washes with PBS, cells were covered with AntiFade and their fluorescent images captured using a Keyence microscope with motorized stage for automated well-by-well imaging, using filters appropriate for DAPI and Thioflavin T. The intensity of Thioflavin T fluorescence per field was quantified via an ImageJ plug-in developed in-house, and normalized to the number of DAPI^+^ nuclei counted per field, to obtain the mean ± SD of aggregate fluorescence per cell for each treatment.

### Western-blotting analysis of ER stress proteins

Proteins isolated from sham-MI, MI, and exosome-treated MI mice were quantified with Bradford reagent (Bio-Rad). Protein aliquots (50 μg) were electrophoresed 2 h at 100 V on a 4‒20% gradient bis–tris acrylamide gel (Bio-Rad Life Science, Hercules, CA), and transferred to nitrocellulose membranes. The membrane was blocked with BSA blocker (Pierce), blots were probed with rabbit antibody to GAPDH (Santa Cruz Biotechnology, 1:1000 dilution), P-PERK (Cell Signaling, 1:1000 dilution) or GRP78 (Abcam, 1:1000 dilution), or ATF6 (Cell Signaling, 1:1000 dilution) overnight at 4 °C. After three washes, membranes were incubated 1-h at room temperature with a secondary antibody: either HRP-conjugated goat anti-rabbit (AbCam, 1:5000 dilution) or rabbit anti-mouse IgG (Rockland Immunochemicals, 1:5000 dilution), developed using an ECL chemiluminescence detection kit (Pierce), and analyzed using ImageJ (NIH).

### Immunostaining and TUNEL assay to detect and quantify apoptosis

Cortex and hippocampus sections (6 samples/group) were treated and processed according to our published protocol [20]. The processed sections were blocked with 5% goat serum/1% BSA in PBS for 30 min at room temperature and then incubated with anti‐GRP78 antibody (1:400, v/v, dilution, Cell Signaling Technology, Danvers, MA) and incubated overnight at 4 °C. The slides were washed three times with PBS and incubated with FITC‐conjugated (Abcam) secondary antibody (1:1000, v/v, dilution) in 3% goat serum/1% BSA for 30 min at room temperature. After washing with PBS, ProlongH Gold antifade reagent with 4,‐diamidino‐2‐phenylindole (DAPI; Life Technologies, Carlsbad, CA) was layered before adding the coverslip and image capture. The treated sections were also used to detect and quantify apoptosis using DeadEnd™ Fluorometric TUNEL kits (Promega Corporation, Madison, WI) following the manufacturer's protocol.

### Functional-annotation clustering meta-analysis by DAVID

DAVID (http://david.abcc.ncifcrf.gov) analyzes lists of differentially expressed genes or differentially abundant proteins by seeking enrichment of functional-annotation terms (also called “gene ontology” or GO terms) associated with each entry in the list, beyond that which would be expected for a random list from the same organism. Functional-annotation clustering eliminates much of the redundancy that arises in GO analyses due in large measure to combining multiple resources for gene or protein annotation. Terms that are associated with the same, or largely overlapping, sets of gene/protein names are presumed to refer to the same biological properties and are therefore clustered together. Outputs are selectable, but include (as in Tables 2 and 3) a name assigned each cluster to represent its biological meaning, *N* (the number of genes associated with any annotation cluster), the fold-change (factor by which the term or cluster is enriched), and the Benjamini-adjusted *P* value of annotation enrichment (using the Benjamini–Hochberg estimate of the false-discovery rate) to correct for multiple but correlated terms analyzed.

### Statistical analysis

Each experimental comparison entailed 3–6 mice per group. Significances of inter-group differences were assessed using 2-tailed heteroscedastic *t* tests, appropriate to groups of unknown or unequal variance. Differences in peptide abundance per sample (i.e., spectral counts relative to the total yield for all peptides) were tested for significance by either chi-squared or Fisher Exact tests, as appropriate for the *N* values per cell.

## Results

### ER stress-response proteins are enriched in heart and brain after MI

Protein folding is facilitated by diverse chaperones and other co-factors, many of which are specific to co-translational misfolding in the ER [26]. The chaperone GRP78 is activated by elevated levels of protein misfolding [26]; it activates all three branches of the ER unfolded protein response (UPR^ER^) and thus serves as a critical sensor of ER stress.

**Fig. 1:**
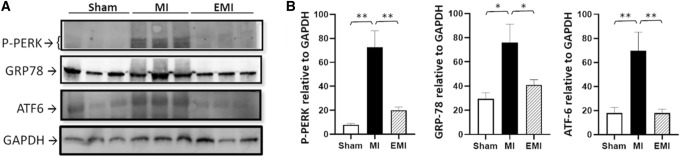
Cardiac ER unfolded protein response (UPR^ER^) proteins are induced by MI but blocked after exosome treatment. Western blots were used to determine relative abundances in cardiac aggregates of 3 proteins that are indicative of UPR^ER^ activity: phospho-PERK (P-PERK), GRP78, and ATF6. **A** Representative western blots are shown for these 3 UPR^ER^ proteins, adding GAPDH as an internal standard for quantitation. **B** Histograms show quantified lane scans, as means ± SD. Significance of differences between MI and other groups was assessed by 2-tailed heteroscedastic *t* tests, appropriate for small groups in which variance is unequal or unknown: *P < 0.05; **P < 0.01

Free or unbound GRP78 suppresses both the activities and the steady-state levels of PERK (Protein kinase-like ER-Kinase) and ATF6 (Activating Transcription Factor 6) [26]. We noted 2.5- to 10-fold increases in protein levels of GRP78, ATF6, and activated/phosphorylated PERK (P-PERK) in ischemic hearts (Fig. 1; *P* < 0.01 or 0.05 vs. sham-MI by 2-tailed heteroscedastic *t* test). Prior administration of MSC exosomes reduced abundance of all three proteins in heart tissue (*P* < 0.05 or 0.01, EMI vs. MI [untreated/saline only] after ligation-induced ischemia, as indicated in Fig. 1).

**Fig. 2:**
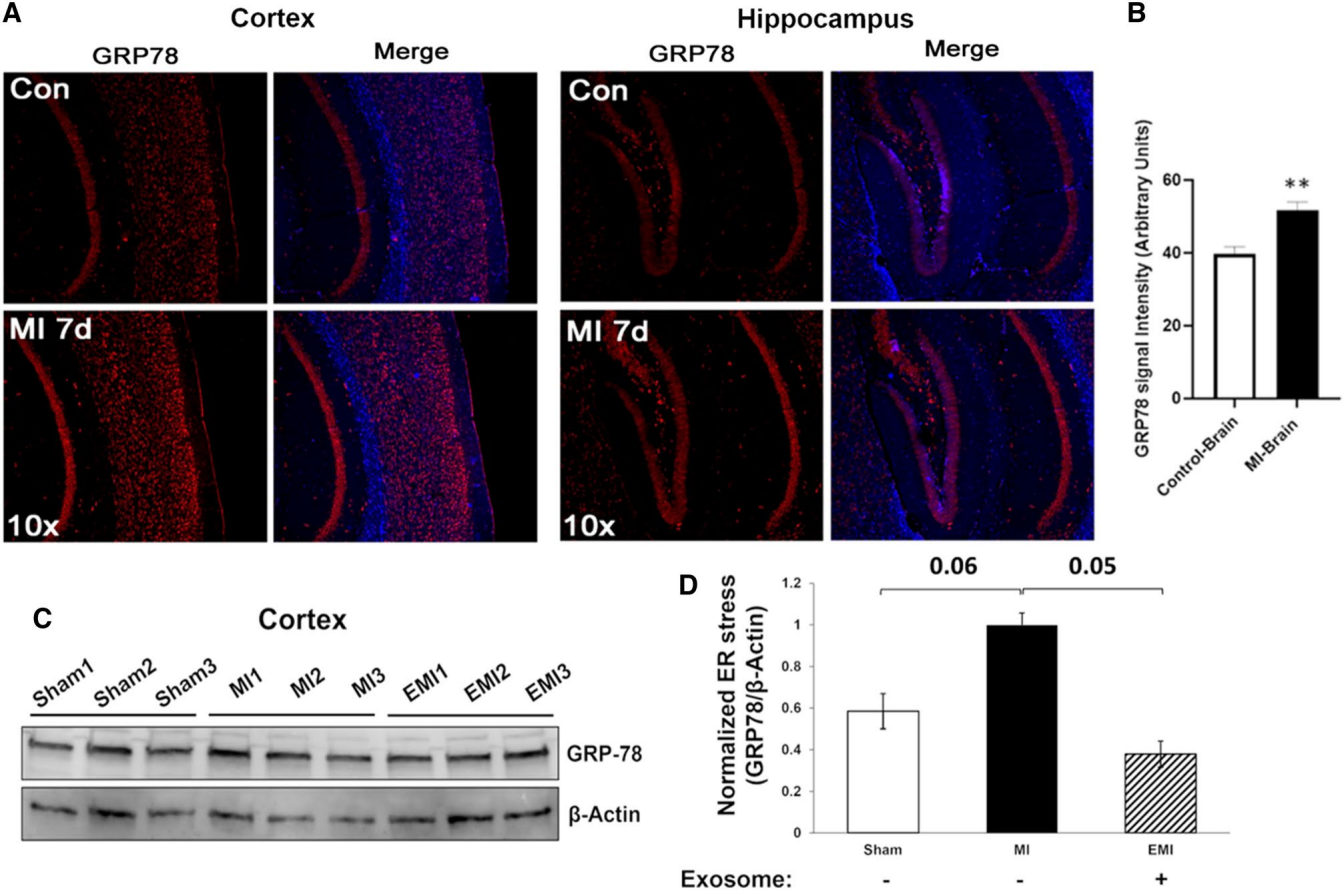
Levels of ER-stress protein GRP78 are elevated in brains of mice that underwent MI vs. sham-MI. **A** Immunohistochemistry (IHC) of cortical and hippocampal brain regions from sham-MI mice (“Con”) and MI mice, 7 days after LCA ligation or sham/control surgery (each *N* = 5). **B** Combined quantitation of both cortex and hippocampal IHC shows significant upregulation of GRP78 (~ 30%) in these brain regions after MI relative to sham-MI (**P < 0.005). **C** Western blot of total protein from sham-MI, MI, and EMI mouse brains, probed with antibodies to GRP78 and β-actin (each *N* = 3). **D** Quantitation of the blot shows GRP78/β-actin expression was elevated in mouse brain after MI relative to sham-MI (*N* = 6; *P* ≈ 0.06) and relative to EMI (*P* ≈ 0.05) by 2-tailed heteroscedastic *t* tests

Cortical and hippocampal brain regions of mice after transient (2-hr) LCA ligation consistently showed GRP78 to be markedly upregulated, as indicated both by immunohistochemistry (Fig. 2A and B; **P ≈ 0.005) and by western blotting (Fig. 2C and D; *P < 0.05) of extracted protein, relative to sham-MI controls. The increase in GRP78 expression was again fully blocked by prior treatment of mice with MSC exosomes (EMI differs from MI, *P ≈ *0.05 by 2-tailed heteroscedastic *t* test; Fig. 2C and D).

Of note, ER stress has been reported to arise from MI and other ischemia-related disease states [27]. Furthermore, brains of patients with AD show elevated ER stress and activation of the ER unfolded protein response (UPR^ER^), accompanied by cytophysiological adaptations that redirect cell fate [27].

### Aggregation increases in heart and brain after MI, with elevation of constituent proteins that are also enriched in Alzheimer’s disease (AD) brain aggregates

**Fig. 3:**
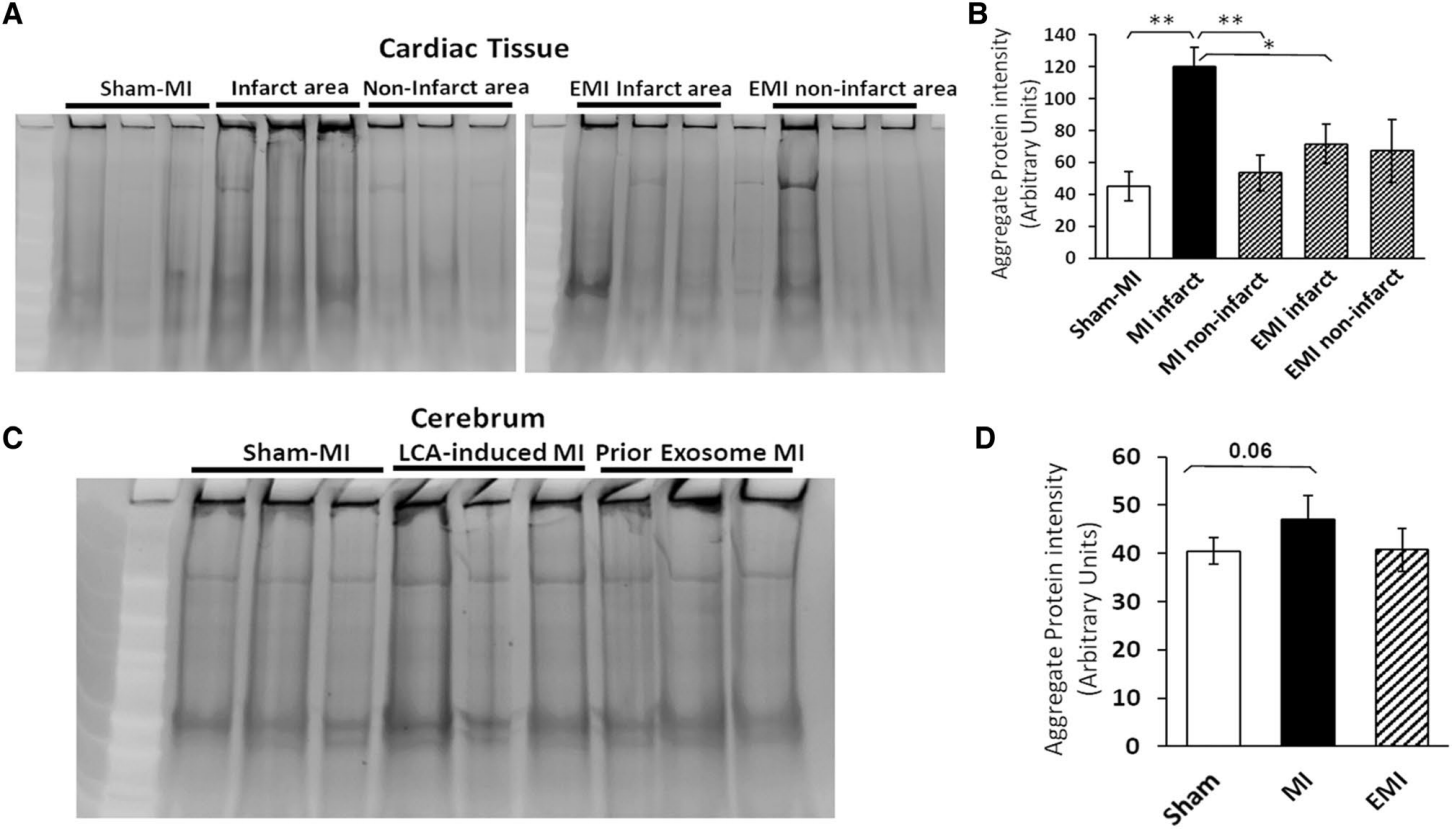
Aggregates increase in hearts and brains of mice after MI. **A**, **B** Infarct areas from MI mouse hearts contain 2.7-fold more detergent-insoluble aggregates than sham-MI heart or peri-infarct heart tissue (**each *P* < 0.01), also exceeding hearts of MI mice pretreated with exosomes (EMI; *P < 0.001). **C**, **D** A smaller increase (15%) that did not achieve significance (*P *≈ 0.06) was observed for insoluble aggregates in brains of MI mice. **A** and **C** Representative gels showing sarkosyl-insoluble aggregates resuspended in Laemmli buffer at 95–100 °C. **B** and **D** Histograms summarize means ± SEM of total fluorescence per lane for 3 mice per group. Significance of inter-group differences was assessed by 2-tailed heteroscedastic *t* tests, appropriate for small groups where variance is unequal or unknown

We previously reported that inflammation is exacerbated by aging and age-associated diseases, all of which feature elevated protein aggregation [5, 6]. Since ER stress leads to increased protein aggregation and is increased after MI, we compared protein aggregation in hearts and brains of mice after MI to hearts and brains of sham-operated mice to assess differences in protein aggregation (Fig. 3A and C). Based on full-lane scans of sarkosyl-insoluble protein stained with SYPRO-Ruby (ThermoFisher), protein aggregation was elevated ~ 2.7-fold in hearts of post-MI mice relative to sham-MI mice (Fig. 3B). The infarcted regions also differed from other (non-infarct) areas of the same post-MI hearts, as well as from sham-MI hearts (*P* < 0.001 by 2-tailed heteroscedastic *t* test). The infarct areas of hearts of exosome-treated mice (EMI) had fewer aggregates than the corresponding heart regions from MI mice (*P* < 0.01), but did not differ from sham-MI heart tissue (Fig. 3B).

We observed a smaller shift toward increased aggregation in the brains of post-MI mice (Fig. 3C and D), a rise of 15% which fell just short of significance (*P *≈ 0.06 by 2-tailed heteroscedastic *t* tests). Of 727 proteins enriched in AD hippocampal aggregates, relative to those from age-matched controls (based on spectral counts) [6], only 29 (4%) were also enriched significantly in cerebral aggregates from post-MI mice (Table 1). Of these 29 proteins with both MI/sham-MI and AD/AMC abundance ratios ≥ 1.5, 16 (55%) were also enriched by ≥ 1.5-fold in MI heart relative to sham-MI (2-tailed Fisher’s Exact test, *P* < 2E−10). These dually differential proteins, listed in Table 1, suggest that similar protein–protein interactions drive aggregation in both tissues. Annotation terms enriched for MI-induced cardiac aggregates, shown in Tables 2 and 3, include mitochondria, cell adhesion, translation, actin binding, RNA binding, TCA cycle, neurodegenerative diseases, and chaperones/protein folding and misfolding.

**Table 1:** Aggregate proteins enriched in Alzheimer’s disease brains are also differentially aggregated after MI in mouse brain and heart

Proteins	AD/AMC (human brain)	MI/Sham (mouse brain)	MI/Sham (mouse heart)
**Short-chain specific acyl-CoA dehydrogenase, mitochondrial (ACADS)**	**10.00**	**1.75**	**3.55**
AP-3 complex subunit mu-2 (AP3M2)	10.00	1.71	ND
Actin-related protein 2/3 complex subunit 2 (ARPC2)	10.00	1.51	ND
**Carbonyl reductase [NADPH] 3 (CBR3)**	**180.00**	**1.60**	**1.71**
**Hsp90 co-chaperone Cdc37 (CDC37)**	**10.00**	**3.23**	**60.00**
**Cytosolic non-specific dipeptidase (CNDP2)**	**7.00**	**1.71**	**120.00**
Lambda-crystallin homolog (CRYL1)	20.00	4.00	ND
Ketimine reductase mu-crystallin (CRYM)	2.00	2.20	ND
Eukaryotic translation initiation factor 3 s.u. K (EIF3K)	10.00	3.42	ND
**Exocyst complex component 4 (EXOC4)**	**1.50**	**1.52**	**160.00**
**Guanine nucleotide-binding protein α-13 (GNA13)**	**2.17**	**2.70**	**2.23**
Hepatocyte growth factor-regulated tyrosine kinase substrate (HGS)	20.00	1.71	ND
Adenosine 5'-monophosphoramidase HINT2 (HINT2)	10.00	1.75	11.00
l-lactate dehydrogenase A chain (LDHA)	1.50	27.33	3.78
Mitochondrial carrier homolog 1 (MTCH1)	1.50	4.00	2.75
Nuclear receptor coactivator 7 (NCOA7)	10.00	7.33	ND
Nebulette (NEBL)	1.50	1.83	2.11
Omega-amidase NIT2 (NIT2)	20.00	2.73	10.00
Protein phosphatase 1E (PPM1E)	10.00	1.71	ND
**Ribose-phosphate pyrophosphokinase 1 (PRPS1)**	**10.00**	**1.94**	**3.00**
E3 ubiquitin-protein ligase RBX1 (RBX1)	10.00	1.50	ND
**Receptor expression-enhancing protein 5 (REEP5)**	**10.00**	**1.95**	**40.00**
Rho-associated protein kinase 1 (ROCK1)	10.00	2.58	ND
**Reticulon-4 (RTN4)**	**2.00**	**1.65**	**2.67**
Sec1 family domain-containing protein 1 (SCFD1)	1.50	13.33	100.00
Protein SGT1 homolog (SUGT1)	3.00	1.75	ND
Tripeptidyl-peptidase 1 (TPP1)	10.00	1.61	ND
Vesicle transport protein USE1 (USE1)	10.00	2.58	ND
**WD repeat-containing protein 1 (WDR1)**	**3.00**	**1.63**	**2.80**

Numbers indicate spectral-count ratios from aggregate proteomics, as indicated. Entries in bold highlight similar changes in AD human brain [6], and mouse post-MI heart and brain

**Table 2:** Cardiac proteins differentially abundant after MI relative to sham-MI

Heart (category)	*N*	Fold change	Benjamini-adjusted *P*
Proteins up in MI vs. sham-MI	1933	3.6	2.2E−11
Proteins up in MI, spared/rescued by EMI	1617	3.3	3.0E−252
Proteins down in MI vs. sham-MI	69	2.0	1.4E−05
Proteins down in MI, spared/rescued by EMI	13	2.0	1.4E−15

Brain (category)	*N*	Fold change	Benjamini-adjusted *P*
Proteins up in MI vs. sham-MI	406	31.8	2.5E−5
Proteins up in MI, spared/rescued by EMI	358	35.1	0.0003
Proteins down in MI vs. sham-MI	206	30.4	0.0005
Proteins down in MI, spared/rescued by EMI	165	35.8	0.0004

Exosome pretreatment alleviates MI-associated aggregation of cardiac proteins

**Table 3:** Pathways enriched in MI-induced insoluble aggregates

GO terms for post-MI heart aggregate proteins (Functional-Annotation Clustering Score, FACS)	*N*	Fold change	Benjamini-adjusted *P*
Mitochondria (FACS = 54.2)	239	3.5	9.5E−69
Cell–cell adhesion (FACS = 31.2)	80	6.4	3.5E−39
Translation; Ribonucleoprotein complex (FACS 39.7)	89	8.4	2.8E−35
Actin binding (FACS = 22.9)	58	6.7	6.5E−28
RNA binding (FACS = 11.4)	82	4.0	8.8E−24
TCA cycle (FACS = 16.1)	47	5.9	4.3E−22
Parkinson’s, Alzheimer’s, Huntington’s (FACS = 12.5)	42	3.4	3.8E−12
Chaperone; protein folding; unfolded protein (FACS = 7.1)	25	4.6	2.1E−6

GO terms for post-MI brain aggregate proteins (Functional-Annotation Clustering Score, FACS)	*N*	Fold change	Benjamini-adjusted *P*
Cell Junction, Synapse (FACS = 9.6)	66	2.6	2.8E−10
Phosphorylation (FACS = 7.6)	48	2.7	4.1 E−6
Mitochondria (FACS = 6.9)	72	2.0	1.2E−6
Pleckstrin homology like domain (FACS = 4.9)	72	2.8	1.9E−4
RNA binding (FACS = 3.7)	58	2.1	2.5E−5
UBL-conjugation (FACS = 4.0)	121	1.4	4.6E−4
Translation; Ribonucleoprotein complex (FACS = 3.6)	14	5.2	2.7E−3
Parkinson’s, Alzheimer’s, Huntington’s dis. (FACS = 2.3)	24	1.9	4.5E−2
Chaperone; protein folding; unfolded protein (FACS = 7.1)	25	4.6	2.1E−6

There were 2002 heart aggregate proteins significantly enriched after MI, 180 (9%) of which had previously shown significant enrichment in aggregates from aged and/or hypertensive hearts [5], suggesting some overlap in the underlying aggregation processes (Supplementary Table 1). These shared aggregate proteins include 14-3-3 paralogs, proteasomal subunits, heat-shock proteins, apolipoproteins, vitronectin, vinculin, filamins, and cardiac phospholamban [5].

### CDN1663 reduces hypoxia-induced ER stress in cultured human glioblastoma cells

**Fig. 4:**
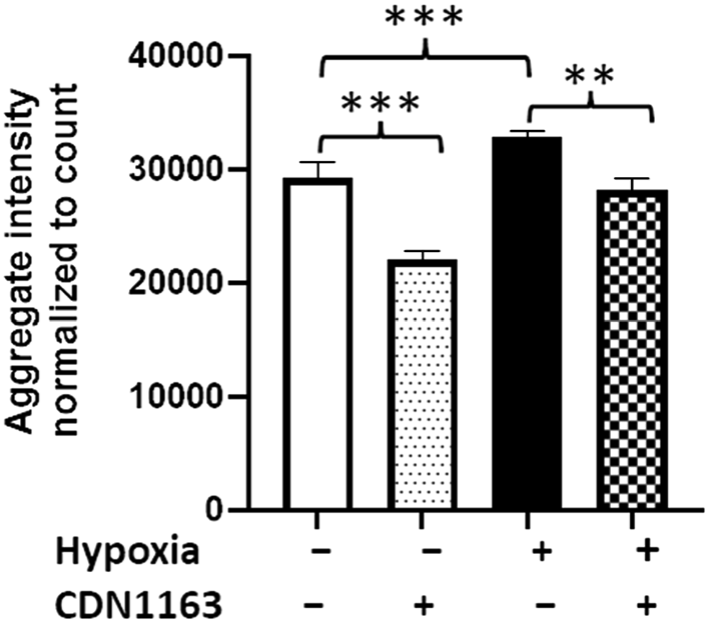
ER stress regulates hypoxia-mediated increases in aggregation. T98G cells were exposed to hypoxia and grown in the presence or absence of 10-µM CDN1163. After 7 h of hypoxia, cells were returned to normoxia and maintained for a further 16 h at 37 °C. Cells were fixed and stained with Thioflavin T. With or without hypoxia, CDN1163 provided significant protection against aggregation (for *N* = 9 biologically independent samples per group), as determined by 2-tailed Student *t* tests. The histogram shows means ± SD. **P < 0.01; ***P < 0.0001

We postulated that MI causes transient hypoxia, inducing ER stress in brain and thus driving protein aggregation. Previous research has demonstrated that ER stress is relieved by the small molecule CDN1163 [28]. We therefore assessed whether CDN1163 treatment of glioblastoma cells ameliorates aggregation arising from hypoxia. To mimic MI, we first exposed human glioblastoma cells (T98G) to hypoxia, leading to a 10% increase in protein aggregation (Fig. 4). Protein aggregation decreased to the normoxic level (a 12% reduction) in hypoxic cells treated with CDN1163 (Fig. 4, *P* < 0.05). Overall, these results are consistent with an elevation in ER stress arising after transient hypoxia, which in turn augments aggregation, whereas relieving ER stress alleviates protein aggregation.

### MI in mice induces cortical-cell apoptosis

**Fig. 5:**
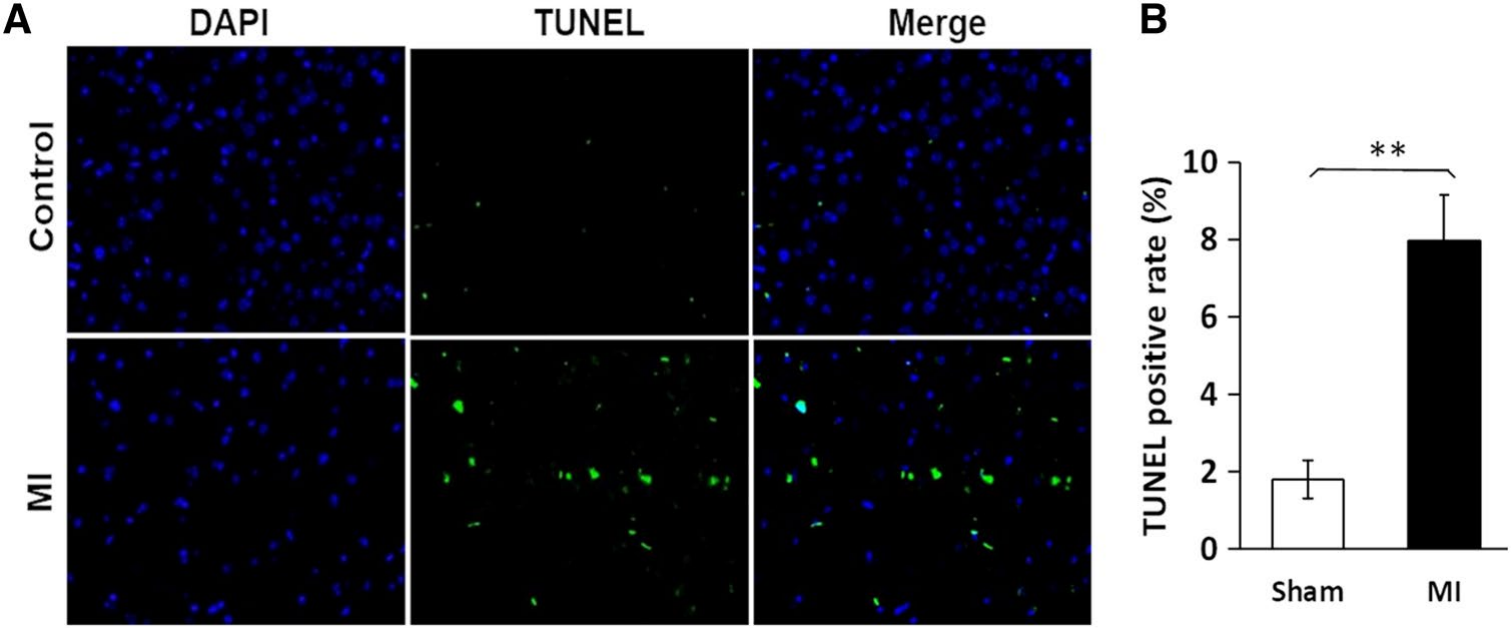
The frequency of apoptosis is elevated fourfold in cerebral (cortex and hippocampus) neurons, relative to sham-MI, 7 days after surgery. **A** TUNEL assay (Promega) images show DAPI-stained nuclei (blue), fluorescein-12-dUTP labeled apoptotic cells (green; identified as neurons by histomorphology) and merged images, typical of control and MI mouse brains. **B** Quantitation of data from 5 mice per group (mean % TUNEL-positive cells, 3 fields per mouse). The apoptosis frequency in MI brain was increased > fourfold after MI relative to sham-MI (**P < 0.0001 by 2-tailed Student’s *t* test)

Apoptosis can be triggered by a variety of stressors, including sustained inflammation, cancer, and environmental stressors [20]. In post-MI mice, cortical-cell apoptosis was quantified by TUNEL (TdT-mediated dUTP nick labeling) using a Promega DeadEnd™  assay as described previously [20]. MI increased the count of apoptotic cortical cells, identified by histomorphology as neurons, by over fourfold (Fig. 5; *P* < 0.0001 by 2-tailed *t *tests). This finding supports our argument that MI increases cytotoxic stressors in the brain, thus triggering neuronal apoptosis.

### Exosomes from hypoxic MSCs reduce MI-mediated protein aggregation

Exosomes are membrane-bound microvesicles released from most or all cells, which carry a complex set of proteins [29]. Exosomes isolated from MSCs are able to reduce MI-mediated fibrosis and to promote regeneration of cardiomyocytes [30]. In the present studies, we report that administration of MSC exosomes reduced post-MI protein aggregation to levels similar to those seen in sham-MI mice (Fig. 3B; *P* < 0.05 by 2-tailed *t* test). Of 2002 proteins that were enriched in heart aggregates after MI, 81% (1617) were partially or fully reversed by exosome pretreatment (Table 2A). Exosomes also appeared to block the MI-mediated increase in brain aggregation (Fig. 3D), and reversed 358 out of 406 proteins (88%) that were enriched in brain aggregates (Table 2). The gene ontology and/or pathway terms most significantly enriched by MI through DAVID functional-annotation clustering analysis (http://david.abcc.ncifcrf.gov) were mitochondria, cell–cell adhesion, translation & ribonucleoprotein complex, actin binding, RNA binding, TCA cycle, neurodegenerative disorders, and chaperone, protein folding, unfolded protein binding (Table 3). Proteins enriched after MI include many that had been previously implicated as components of protein homeostasis machinery, mitochondrial energy metabolism, and associated with neurodegenerative and/or cardiovascular disease (Supplemental Table 1).

## Discussion

The incidence of MI is elevated by a variety of age-associated risk factors including diabetes and hypertension, as well as age itself—apparently mediated via “inflammaging” [31] and impaired protein homeostasis. Recent studies have shown that autophagy pathways, required for clearance of aggregates, are compromised in individuals following MI [12]. A phospholamban gene variant was reported to alter proteostasis, elevating phospholamban aggregation and predisposing to cardiomyopathy [32]. However, the relationship between MI and protein misfolding or aggregation is not well understood. Here, we demonstrate that MI increases ER stress and detergent-insoluble aggregates in both heart and brain. Moreover, cardiac-aggregate proteins differentially enriched after MI overlap substantially with those we previously identified as enriched in mouse hearts by hypertension and aging [5, 6] and in human brain due to AD [5, 6]. The ability of exosomes from mesenchymal stem cells to alleviate MI-induced protein aggregation in MI hearts may open the door for novel therapeutic approaches.

The endoplasmic reticulum (ER) is known to play critical roles in protein homeostasis, because many proteins released into the ER during translation undergo initial (co-translational) misfolding there, abetted by ribosomal and ER chaperones prior to secretion. ER stress is a primary indicator of insufficient proteostasis; it is elevated by diverse stresses, and is accompanied by increases in ER-stress-sensor chaperones such as GRP78, which binds both the ER transmembrane protein kinase PERK, and transcription factor ATF6 which translocates to the nucleus to drive expression of other ER-stress-responsive chaperones [26]. Both PERK and ATF6 are activated by GRP78 in response to UPR^ER^ [26]. Here, we show increased ER stress (as indicated by levels of PERK, GRP78, and ATF4 proteins) in hearts, and more importantly in cerebra, of mice after experimentally induced MI. Several proteins critical to proteostasis, including 26S proteasomal subunits, chaperonin HSP60, and autophagy mediator TRIM28, are also enriched in post-MI heart aggregates. Transcriptional corepressor TRIM28 (tripartite motif-containing protein 28), enriched sevenfold in cardiac aggregates after MI (see Supplementary Table 1), was shown to regulate autophagy in cancer cells [33]. We also observed elevated protein aggregation in glial cells under hypoxic conditions, an increase that is reversed by treatment with a small molecule known to relieve ER stress, CDN1163. Our study demonstrates that elevated ER stress, the ubiquitin/proteasome system, and autophagy are all compromised after MI, leading to proteostasis failure and elevated aggregation.

Heart proteins markedly induced by MI include filamins A and B, clusterin (ApoJ), cardiac phospholamban, and NEDD4. Filamins A and B are actin-binding proteins expressed in endothelial cells (see Supp. Table 1). Filamin B was previously determined to play roles in vascular endothelial growth factor (VEGF)-induced endothelial cell motility and in angiogenesis [34]. Cardiac phospholamban is a major regulator of cardiac cell contractility. In its dephosphorylated state, it modulates calcium sequestering by inhibiting the action of Sarcoplasmic Reticulum Calcium ATPase (SERCA) [35]. In disease conditions such as heart failure, dephosphorylated phospholamban leads to depressed calcium cycling and impaired calcium transport. When phospholamban becomes phosphorylated by protein kinase A, it blocks SERCA inhibition and thus augments SERCA activity [35]. We observed increased sequestration of both phospholamban and SERCA in our aggregate proteomics data (Supplementary Table 1). Transthyretin (TTR), a plasma protein synthesized in the liver, is involved in transport of retinol and the thyroid hormone thyroxine. Its insoluble deposition in heart muscle has been shown to cause Transthyretin Cardiac Amyloidosis (ATTR-CA) [36].

**Fig. 6:**
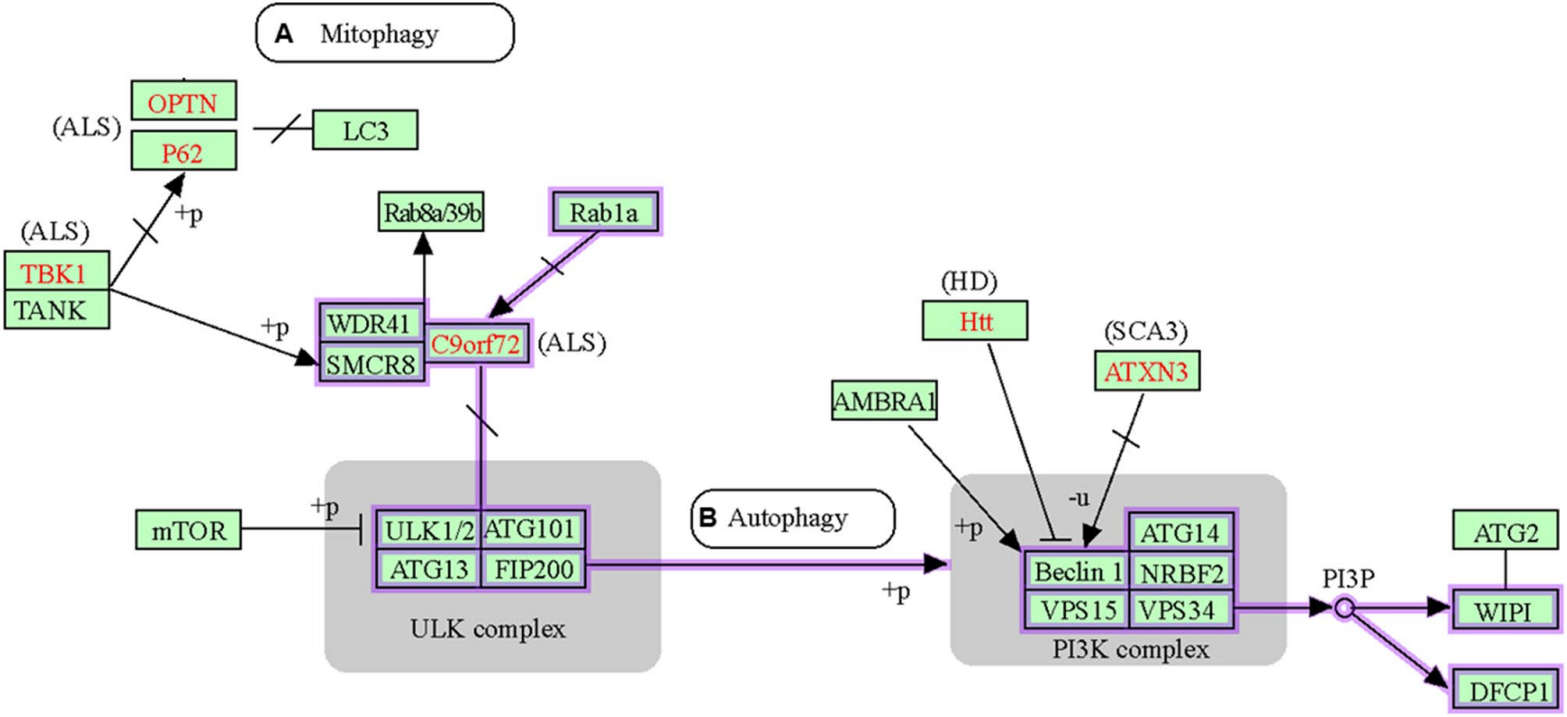
DAVID analysis of protein annotation terms (highlighted with purple rectangles) that are enriched for proteins in aggregates arising post-MI, in both hearts and cerebra of LCA-ligated mice. **A** Proteins involved in mitophagy.** B** Proteins involved in autophagy. Pathway figures were created within DAVID (http://david.abcc.ncifcrf.gov) using KEGG annotations

To gain insights into the biological roles of proteins differentially aggregated after MI, we performed gene ontology and pathway meta-analysis (https://david.ncifcrf.gov). Proteins enriched in post-MI cardiac aggregates showed the highest significance for annotation terms mitochondria, cell adhesion, translation, actin binding, RNA binding, protein folding, TCA cycle, and three neurodegenerative diseases (AD, Parkinson’s disease, and Huntington’s disease) (Fig. 6, Supp. Fig. 1, and Table 3). Several of these terms support the argument that proteostasis failure is a predisposing condition for cardiovascular disease, just as it is for neurodegenerative diseases. The observation that over half of the proteins (16 of 29) enriched by at least 50% in both AD and MI brains (relative to AMC and sham-MI respectively) were also enriched in mouse hearts after MI (Table 1) likely reflects the increased propensity of disordered proteins (those with extensive regions of intrinsic disorder) to enter into aggregation complexes. These data suggest that similar declines in protein homeostasis occur in both heart and brain, in response to aging and/or oxidative stress due to hypoxia/reperfusion. Although many differences exist—as expected given the tissue-specificity of protein expression—the similarities between heart and brain aggregates, accompanying age-associated proteostasis failure, support the suggestion that MI is “Alzheimer’s disease of the heart” [37].

This study extends our previous work demonstrating that protein aggregation increases in hearts of aged and hypertensive mice [5], demonstrating commonalities between ischemic heart disease and hypertensive and aged hearts. A subset of proteins enriched in aggregates from MI mice (~ 9% of such proteins) mirror those enriched in heart aggregates from hypertensive mice [5]. These include von Willebrand factor (previously implicated in CVD), TDP-43 (a protein diagnostic for ALS aggregates), Apolipoproteins A–I and A–IV (serum markers/risk factors for CVD), transthyretin (implicated in both AD and CVD), filamins A and B (implicated in cardiac development), and a variety of microtubule-associated proteins (Supplementary Table 1). Indeed, of the top 20 proteins strongly induced by MI (> fourfold), 12 (60%) were also elevated at least twofold by hypertension [5], and aged hearts also show an overlap of 11 proteins (55%; Supplementary Table 1).

This study also shows parallels in protein aggregation between MI hearts and neuropathologies such as Alzheimer’s disease, Parkinson’s disease, and Amyotrophic Lateral Sclerosis (ALS). Aggregate proteins that are significantly elevated in MI brains and shared in common with MI heart aggregates are also enriched in the aggregates of AD brains (see Table 1 and Supplementary Table 1).

A striking feature of MI-induced changes in brain aggregates is the enrichment of 29 proteins also elevated in Alzheimer’s disease brains (see Table 1 and [6]). These post-MI changes in brain aggregates may lead to behavioral symptoms including depression and anxiety, seen after MI in many patients [38].

An important translational finding in the current study is the therapeutic potential of MSC exosomes to blunt MI-mediated protein aggregation. Exosomes play key roles in intercellular communication [29], affecting cell biology, cancer, cardiovascular disease, and neuronal stress responses. They can travel though the circulation to remote sites, and are able to cross the blood–brain barrier [39]. We previously reported that the infarct size was reduced after exosome treatment [30]. Our observation in this study that MSC exosomes oppose the pro-aggregation effects of myocardial ischemia implies that they also play vital cardioprotective roles. All major cardiac cell types (including cardiomyocytes, endothelial cells, and fibroblasts) release exosomes that modulate cell functions [19]. Proteomic studies comparing MI, sham-MI, and exosome-pretreated MI mouse hearts, demonstrate remarkable cardioprotection when exosomes are injected into mice prior to myocardial ischemia, effectively blocking the ensuing protein aggregation. The exosomes used in this study, shed by MSCs grown under hypoxic conditions, were injected into mice via the tail vein. These experiments provide proof of concept that cardioprotective therapies have the potential to prevent aggregation in post-MI hearts and brains. Further research is needed to identify exosome constituents that confer protection against ischemic injury, which may lead to the discovery of novel therapeutic targets and agents.

**Fig. 7:**
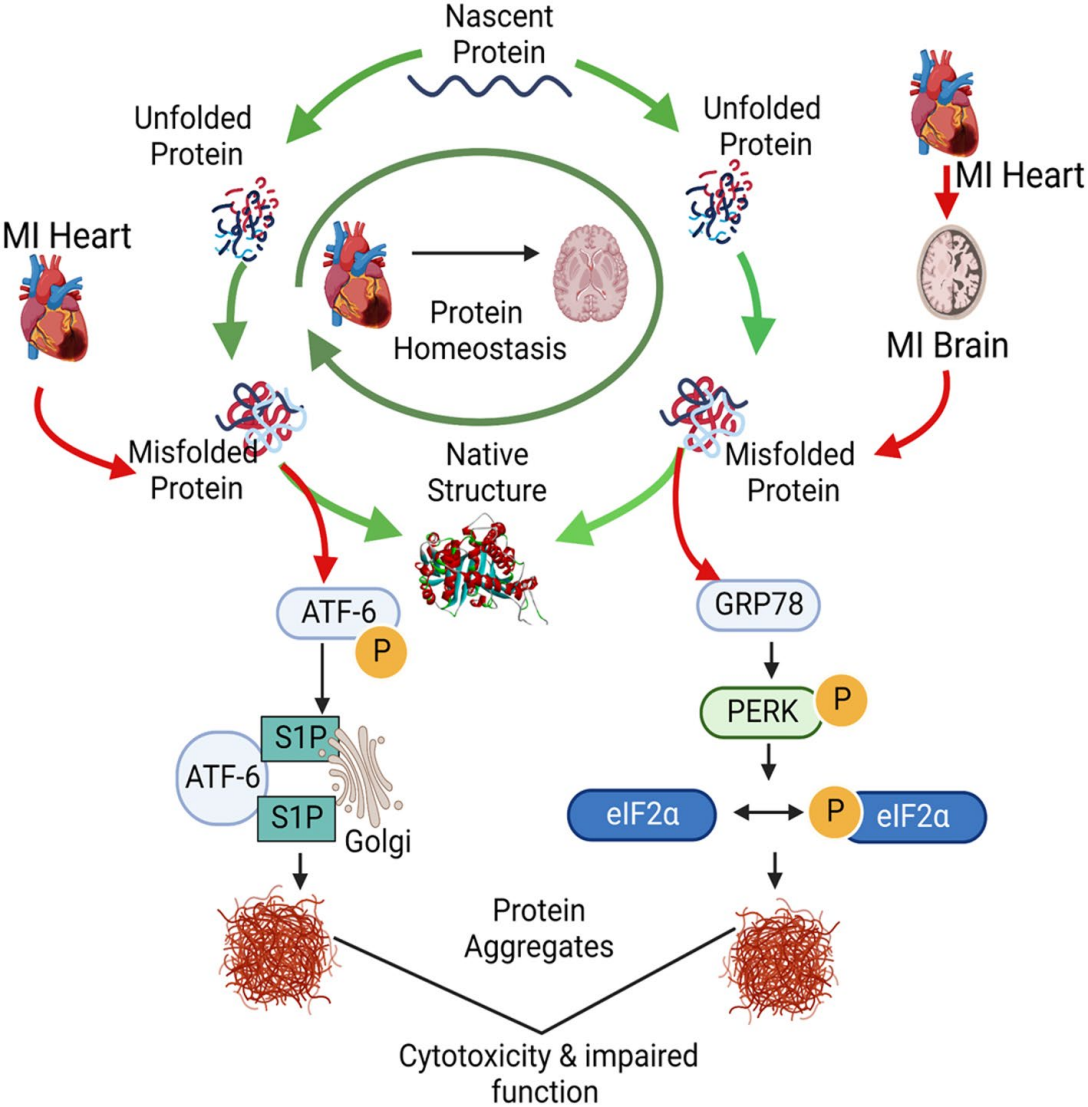
MI-mediated ER stress increases protein aggregation in heart and brain, causing AD-like protein aggregation. This figure provides a schematic illustration of processes after MI that parallel AD in inducing ER stress, protein misfolding, and aggregation in affected tissues, ultimately resulting in cytotoxicity and impaired function. Protein aggregation is a shared mechanism believed to mediate both cytotoxicity and impaired functions of affected tissues and organs

The present study has shown that MI elevates protein aggregation not only in hearts but also in the brains of mice following transient ischemia. Aggregate constituents shared by heart and brain, and overlapping with aggregate proteins enriched in AD hippocampus [6], suggest that misfolding of vulnerable proteins may contribute to the progressive elevation of ER stress and the increase in AD risk after MI, especially considering that both are likely to be mediated by aggregation (Fig. 7), and are strongly associated with apoptosis [40].

These observations implicate key aggregate proteins as potential targets for aggregate attenuation, which could spare both cardiac and brain functions (Fig. 7). The reversal of ER stress and protein aggregation by MSC exosomes opens up novel therapeutic avenues to alleviate myocardial ischemia and reduce the risk of subsequent cognitive decline.

## Supplementary Information

Below is the link to the electronic supplementary material.

Supplemental Figure 1: DAVID analysis of protein annotation terms that are enriched for proteins in aggregates arising post-MI, in both hearts and cerebra of LCA-ligated mice (terms highlighted with purple rectangles). (**A**) Proteins involved in the Ubiquitin-Proteasome System (UPS). (**B**) Proteins involved in the Unfolded Protein Response. (**C**) Proteins involved in apoptosis. (**D**) Proteins involved in neurodegenerative diseases. These figures were created by KEGG and DAVID (http://david.abcc.ncifcrf.gov). (TIFF 1369 KB)

Supplementary file2 (PNG 1043 KB)

Supplementary file3 (XLSX 17 KB)

Supplementary file4 (XLSX 14 KB)

## Data Availability

All data will be made available upon request to the authors.
