# Supplementary figures and images for: Myocardial infarction elevates endoplasmic reticulum stress and protein aggregation in heart as well as brain
Source: Mol Cell Biochem. 2023 Nov 3;479(10):2741–53. doi: 10.1007/s11010-023-04856-3 (PMC11455681; doi:10.1007/s11010-023-04856-3)

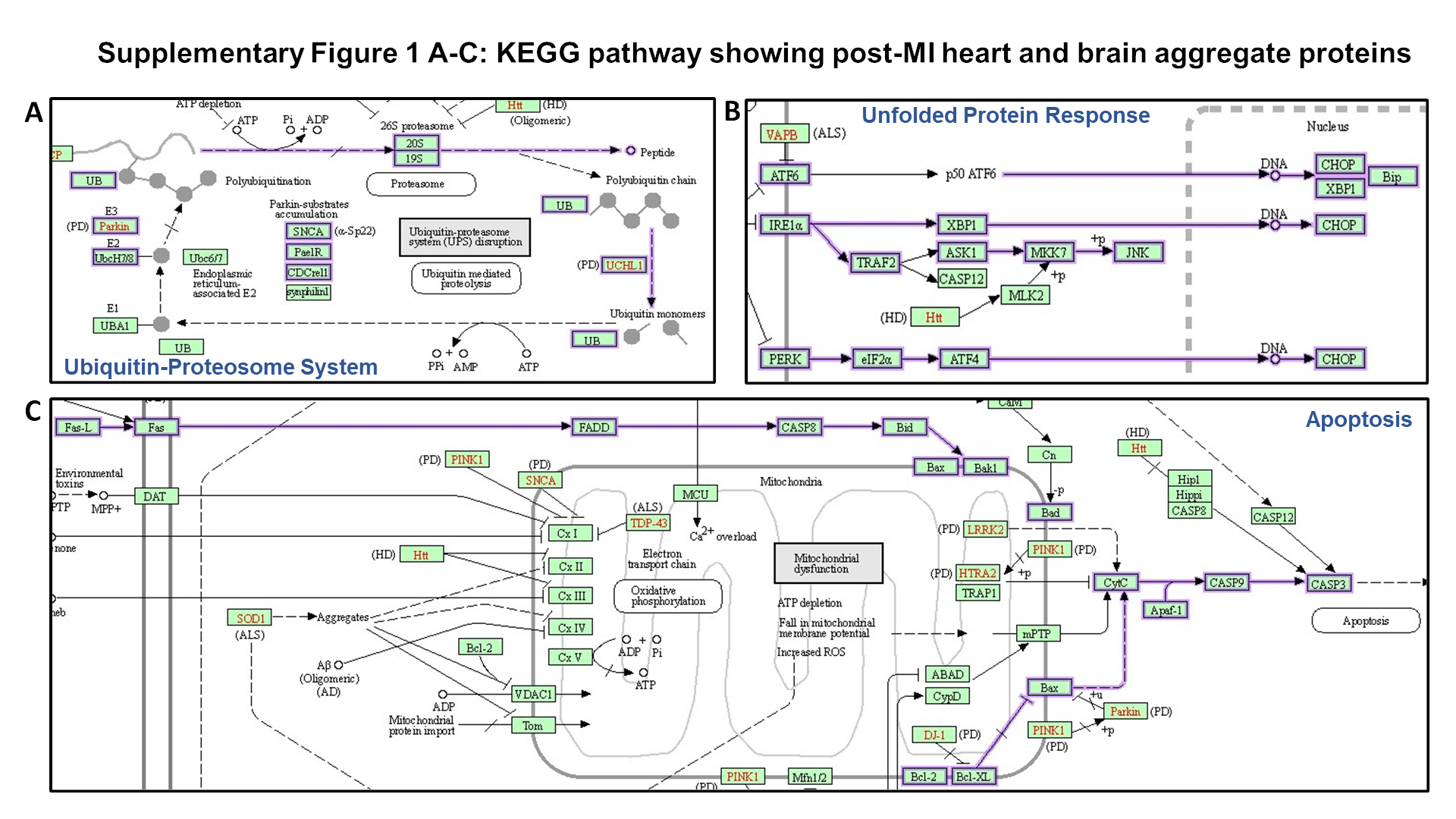

Supplement: Supplementary file 1 — Supplemental Figure 1: DAVID analysis of protein annotation terms that are enriched for proteins in aggregates arising post-MI, in both hearts and cerebra of LCA-ligated mice (terms highlighted with purple rectangles). (A) Proteins involved in the Ubiquitin-Proteasome System (UPS). (B) Proteins involved in the Unfolded Protein Response. (C) Proteins involved in apoptosis. (D) Proteins involved in neurodegenerative diseases. These figures were created by KEGG and DAVID (http://david.abcc.ncifcrf.gov). (TIFF 1369 KB) [file 11010_2023_4856_MOESM1_ESM.tiff]

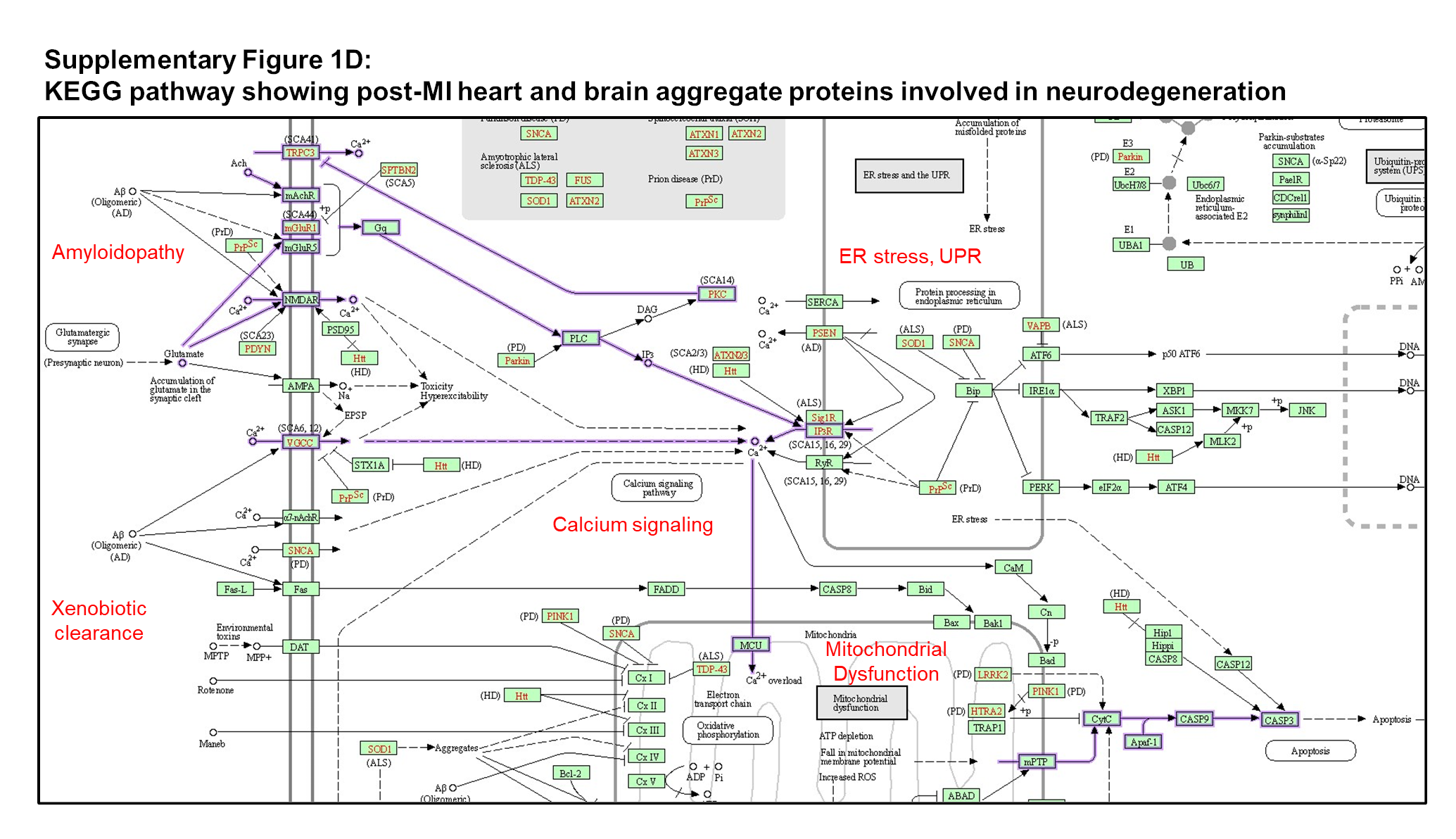

Supplement: Supplementary file 2 — Supplementary file2 (PNG 1043 KB) [file 11010_2023_4856_MOESM2_ESM.png]
